# Editorial: Motivation-Cognition Interaction: From Neurocognitive Models to Clinical Applications

**DOI:** 10.3389/fpsyg.2021.684586

**Published:** 2021-04-28

**Authors:** Elisa Di Rosa, Todd Braver, Daniela Mapelli, Nicky Edelstyn

**Affiliations:** ^1^Department of General Psychology, University of Padua, Padua, Italy; ^2^Department of Psychological and Brain Sciences, Washington University in St. Louis, St. Louis, MO, United States; ^3^School of Psychology, Keele University, Staffordshire, United Kingdom

**Keywords:** motivation, reward, aging, psychiatry, clinical neuropsychology, clinical psychology, cognitive neuroscience

In a recent definition (Botvinick and Braver, 2015), motivational-cognition interactions have been defined in terms of “the invigorating impact, on both behavior and cognition, of prospective reward (both extrinsic reward, such as money, and intrinsic reward tied to the satisfaction of self-relevant behavioral goals, and including negative rewards, i.e., punishments)” (Botvinick and Braver, 2015). This definition captures the idea that incentives, both positive and negative, consequently induce motivational states, which in turn lead to dynamic changes in cognitive processing and therefore behavior.

The last two decades have witnessed a sharp rise in the number of publications on the topic of motivation-cognition interactions. One reason for this increase is the availability of human functional neuroimaging methods (such as fMRI, PET, and fNIRS), which have led to an improved understanding of the neural correlates of motivation-cognition interaction mechanisms. Specifically, these methods have suggested the critical role played by dopaminergic modulation of both cortical and subcortical structures, especially the prefrontal cortex, the anterior cingulate and the parietal cortex (Westbrook and Braver, 2016). Another contributing factor is the growing awareness of the potential role of dysfunctional motivation-cognition interaction mechanisms in the development of abnormal behaviors in several clinical conditions, such as Parkinson's disease, Schizophrenia, and Eating Disorders. Indeed, the study of these and others clinical conditions can also illuminate understanding of healthy brain function.

This Special Research Topic covers several of the different paradigms, methodologies and conditions that have been brought to bear on motivation-cognition interactions, from cognitive neuroscience, cognitive and clinical neuropsychology, to clinical psychology and psychiatry. The aim of this collection is to put together the newest evidence that has been emerging from this exciting research field, which highlights the important link between basic and clinical research, and ultimately points to the invaluable role of an interdisciplinary approach. The articles that have been collected under this topic comprise 14 contributions, which include 11 original research articles and 3 brief research reports. We encourage interested researchers to give the collected articles a thorough reading, in order to gain a complete understanding of how basic neurocognitive models of motivation-cognition interaction can be directed toward different clinical applications.

A first section of papers presents new research on healthy participants. Many of these papers yield important new insights into the nature of motivation-cognition interactions. Massar et al. provide evidence regarding the importance of considering both task type and effort levels, when assessing punishment (loss aversion) effects on cognitive performance. Likewise, Crawford et al. suggest the importance of considering the type of incentive, in that they found robust effects of both monetary and liquid incentives on cognitive performance and self-reported affect and motivation, but weaker motivational and affective effects for social incentives. A number of papers compared motivation-cognition interactions among younger and older adults. Interestingly, Jang et al. provided evidence for the demotivating/distracting effects of loss incentives, respectively, in the two age-groups. Bowen et al. also tested both younger and older adults, but focused on reward anticipation, to demonstrate that these incentives bolster memory in a relatively automatic, rather than strategic, fashion. In contrast, Di Rosa et al. highlight the role of anxiety among both age groups, in producing potential distracting effects of reward motivational incentives. Le et al. utilized functional neuroimaging (fMRI) to examine the neural basis of aging effects on reward motivation, observing clear evidence of age-related reductions in activity in a rewarded Go/NoGo task, that mediated the observed behavioral changes. Zhuang et al. focused on intra-individual variability, demonstrating that hormonal changes during the menstrual cycle influenced impulsivity and both activity and connectivity in frontostriatal circuits. Schiff et al. also examined intra-individual variability, finding a significant effect of fasting on the motivational modulation, by food-reward stimuli, of attentional and cognitive control mechanisms. Taken together, this first set of papers demonstrate the need to consider variables that have previously received less attention in motivation studies, such as the kind of motivational incentive being (social vs. monetary vs. food; positive vs. negative), inter-individual variability in psychological variables such as trait anxiety, and as well non-psychological intra-individual variables, such as hormonal levels or the hours from the last meal. Additionally, these papers clearly show the importance of investigating how aging can impact these mechanisms, since younger and older adults have been found to respond differently to different kinds of incentive manipulations.

A second set of papers illustrate the utility of applying motivation-cognition interaction models to the study of clinical populations. Two studies were conducted on eating disorders. In Chami et al. the researchers demonstrated the efficacy of food-specific inhibitory control training, in patients with bulimia nervosa and binge eating disorder, using an innovative paradigm based on principles related to motivation-cognitive control interaction mechanisms. In Cardi et al. the researchers analyzed the role of motivation in a self-help intervention for patients with anorexia nervosa, showing its role in predicting drop-outs, alliance with the therapist, psychological distress and the ability to change. Two studies provided new evidence on motivation-cognition mechanisms in schizophrenia. Kreis et al. reported the presence of a reduced effort investment in patients with schizophrenia, but also indicated the lack of a direct link between objective and subjective measures of effort. ten Velden Hegelstad et al. did not find any relation between motivation and memory performance in patients with psychosis, suggesting that the primary deficit may be cognitive rather than motivational in nature. Taken together, these two works clearly indicate the need to additional investigate this clinical condition, and moreover highlight the need of further investigation about the feasibility of using subjective vs. objective measures of effort. Last but not least, two original contributions concern the study of patients with mild cognitive impairment (MCI) and with Alzheimer's disease (AD). Using resting state fMRI, Wang et al. examined the effect of a music-based intervention on the intrinsic connectivity of the auditory and the reward neural systems, reporting on the presence of dysfunctional within and between-network connectivity in AD patients, when compared with individual with MCI and with healthy controls. Yin et al. analyzed data from the Chinese Longitudinal Healthy Longevity Survey, reporting a significant association between social support and rewarding activities, like children's visit, in reducing the risk of cognitive impairment among older adults.

To conclude, we are pleased to note that the Special Research Topic offers a diverse set of fourteen engaging papers, each of which addresses in its own way the theme of this issue: Motivation-Cognition interaction. We thank the Frontiers in Psychology Editorial team for their commitment to the project over the past months, the many reviewers who kindly helped us, the researchers whose work is published here and, most importantly, the participants who took part in their studies.

## Author Contributions

All authors listed have made a substantial, direct and intellectual contribution to the work, and approved it for publication.

## Conflict of Interest

The authors declare that the research was conducted in the absence of any commercial or financial relationships that could be construed as a potential conflict of interest.
